# Artificial Intelligence and Eye Disease—Bridging Innovation and Clinical Practice

**DOI:** 10.3390/jcm15166237

**Published:** 2026-08-12

**Authors:** Haoyu Chen, Louis Arnould, Andrzej Grzybowski

**Affiliations:** 1 Department of Ophthalmology and Visual Sciences, The Chinese University of Hong Kong, Hong Kong, China; haoyuchen@cuhk.edu.hk; 2 Joint Shantou International Eye Center, Shantou University & The Chinese University of Hong Kong, Shantou 515041, China; 3 Ophthalmology Department, University Hospital Dijon, 21000 Dijon, France; 4 Department of Ophthalmology, University of Warmia and Mazury, 10-561 Olsztyn, Poland; 5 Institute for Research in Ophthalmology, Foundation for Ophthalmology Development, 60-836 Poznan, Poland

The integration of artificial intelligence (AI) into ophthalmology represents one of the most transformative technological developments in modern medicine [1]. As Guest Editors of this Special Issue, we have witnessed a remarkable convergence of computational innovation and clinical demand, resulting in tools that are reshaping how eye diseases are detected, monitored, and managed. The nine contributions assembled in this issue collectively highlight both the vast potential of AI in ophthalmic care and the challenges that must be addressed before these technologies can achieve widespread and sustainable clinical adoption.

## 1. From Screening to Precision Diagnosis

A recurring theme throughout this collection is AI’s ability to enhance diagnostic accuracy and efficiency across a broad range of ocular conditions. The application of deep learning to fundus photography has matured substantially, as demonstrated by Karabeg and colleagues in their pilot study comparing automated AI grading with manual consensus grading for diabetic retinopathy (DR) screening in Oslo [2]. The EyeArt system achieved a sensitivity of 94.0% for any DR and 89.7% for referable DR, supporting the feasibility of AI-assisted screening in real-world clinical settings. However, the relatively modest specificity for any DR (72.6%) highlights an important limitation. As the authors appropriately emphasize, maintaining robust human grading expertise remains essential for generating high-quality reference standards and ensuring continued algorithm refinement.

This balance between algorithmic promise and real-world performance is reflected in the broader literature [3]. A systematic review and meta-analysis by Wang and colleagues confirmed the high diagnostic accuracy of multiple deep learning systems for DR screening while demonstrating substantial heterogeneity across studies [4]. Likewise, the ACCESS randomized controlled trial showed that autonomous, AI-based diabetic eye examinations performed at the point of care dramatically improved screening completion rates among young people with diabetes (100% versus 22% in the control group) while maintaining appropriate follow-up for abnormal findings [5]. Huang and colleagues further demonstrated that autonomous AI systems can improve access to diabetic eye care and promote health equity in underserved populations [6]. Together, these studies suggest that the field has progressed beyond proof of concept. The central question is no longer whether AI can accurately identify DR, but how it can be effectively and equitably integrated into diverse healthcare systems.

The diagnostic frontier extends well beyond retinal vascular disease. Samoilă and colleagues provided a systematic review of AI applications in papilledema detection, showing that convolutional neural networks can achieve sensitivity and specificity comparable to, and in some cases exceeding, expert-level fundoscopy [7]. Such advances have important implications for telemedicine and early diagnosis, particularly in regions where neuro-ophthalmology expertise is limited.

Similarly, Saito and colleagues introduced the Hokkaido University Pachychoroid Index (HUPI), a deep learning-derived metric capable of quantifying pachychoroid features from enhanced-depth imaging optical coherence tomography (EDI-OCT) [8]. Their findings reveal distinct phenotypic differences among neovascular age-related macular degeneration (nAMD) subtypes, with type 1 macular neovascularization and polypoidal choroidal vasculopathy exhibiting more pachychoroid-like characteristics than type 2 lesions. Beyond improving disease characterization, this work exemplifies how AI can uncover clinically meaningful patterns that may be difficult to identify using conventional assessment methods.

## 2. Predictive Analytics and Personalized Treatment

Perhaps the most transformative applications of AI lie not in diagnosis alone but in prediction and personalized care. The DMEK Risk and Outcome Prediction (DROP) Score developed by Işık and colleagues exemplifies this evolution [9]. By integrating patient characteristics, donor tissue quality, surgical complexity variables, and center-specific performance indicators within a machine learning framework, the DROP Score enables individualized prediction of outcomes following Descemet membrane endothelial keratoplasty. The identification of diabetes mellitus as the strongest prognostic factor (OR 4.34), together with the observation that high-risk patients experienced significantly poorer 12-month best-corrected visual acuity, illustrates how AI can move beyond pattern recognition toward clinically actionable prognostic modeling. Such tools have the potential to enhance surgical counseling, optimize patient selection, and support shared decision-making.

## 3. Structural Analysis and Surgical Planning

In a similar manner, anterior segment imaging has benefited from rapid advances in AI. The multi-granularity mask-guided network (MMNet), developed for automated LOCS III cataract grading using anterior-segment OCT images, represents a notable technical achievement [10]. The system achieved AUC values ranging from 0.954 to 0.973 for cortical, nuclear, and posterior subcapsular cataracts while requiring only 0.0178 s per image for grading. Such performance suggests that AI may soon assume routine grading tasks, allowing clinicians to devote more time to complex clinical decision-making. Importantly, the integration of lesion segmentation and severity classification within a unified framework demonstrates how modern AI architectures can simultaneously address multiple clinically relevant tasks. Beyond diagnosis, automated cataract grading may assist preoperative planning by facilitating the selection of surgical techniques and technologies tailored to cataract severity.

In the posterior segment, vitreomacular interface disorders are common conditions that frequently require surgical intervention [11]. Durmaz Engin and colleagues compared expert-designed deep learning models with automated machine learning (AutoML) platforms for the classification of vitreomacular interface disorders using OCT images [12]. Although the expert-designed model achieved superior balanced accuracy (95.97%), the accessibility of AutoML platforms to clinicians without coding expertise highlights an important consideration for democratizing AI development. The balance between peak performance and ease of implementation is likely to influence the future adoption of many AI-based ophthalmic solutions.

Another area of growing interest is the analysis of the foveal avascular zone (FAZ) using optical coherence tomography angiography (OCTA) [13,14]. Pighin and colleagues compared two methods for measuring FAZ parameters before and after phacoemulsification surgery and found that a machine learning-based approach performed comparably to semiautomated script-based algorithms [15]. However, important methodological considerations remain. The study did not report OCTA image quality metrics, which are known to significantly influence FAZ measurements [16] nor did it evaluate the impact of different OCTA algorithms on quantitative assessment [17]. These factors should be considered in future investigations to ensure robust and reproducible AI-assisted measurements.

## 4. Workflow Integration and the Human Element

Technological sophistication, however, does not guarantee clinical adoption. Tappeiner’s survey of Swiss ophthalmologists and residents reveals a striking paradox: while willingness to use AI exceeds 65% across diagnostic scenarios, actual clinical use remains below 12.1%. The barriers are systemic rather than technical—only 6.6% of respondents reported institutional AI guidelines, and merely 15.1% felt adequately prepared through education. Critically, 93.9% endorsed AI in an assistive rather than autonomous role, reflecting a profession that values human oversight [18]. The message is clear: algorithmic performance must be matched by institutional readiness, regulatory clarity, and structured educational curricula.

This tension between capability and implementation is further illustrated by Alsumait and colleagues’ evaluation of ChatGPT-4 for triaging patient messages in an eye clinic. While GPT-4 demonstrated reasonable agreement with an ophthalmologist-in-training for triage acuity (Cohen’s kappa = 0.67), its 64.7% agreement for specialty clinic direction and tendency to over-triage (recommending equal or sooner evaluation in 93.5% of cases) highlight the risks of unsupervised deployment [19]. The authors’ conclusion—that AI should complement rather than replace physician judgment—resonates as a guiding principle for the field.

The broader literature on large language models in medicine, including the seminal work by Thirunavukarasu et al., demonstrates that these models are approaching expert-level clinical knowledge and reasoning in ophthalmology [20]. However, as our own Special Issue contributions and external studies consistently show, the gap between algorithmic capability and responsible clinical integration remains substantial. Addressing this gap requires not merely better algorithms but better infrastructure, clearer governance frameworks, and purposeful education that prepares clinicians to be informed users rather than passive recipients of AI recommendations.

## 5. Regulatory Landscape and the Path Forward

The regulatory environment for AI in ophthalmology is evolving rapidly. Grzybowski’s recent analysis of CE-certified AI medical devices for DR screening in the European Union identified over a dozen commercially available systems, ranging from autonomous platforms, like IDx-DR and RetinoScan, to assistive tools, like RetCAD and Retmarker DR [21]. This regulatory maturation is essential for clinical adoption, yet it also introduces new complexities. The forthcoming EU AI Act will impose additional transparency and fairness requirements, while post-market surveillance obligations under the Medical Device Regulation demand continuous performance monitoring. The diversity of regulatory pathways across jurisdictions—FDA clearance in the United States, CE marking in Europe, and national approvals elsewhere—creates a fragmented landscape that manufacturers and clinicians must navigate.

## 6. Conclusions

The studies presented in this Special Issue, together with recent advances reported throughout the broader ophthalmic literature, demonstrate that AI has progressed from proof-of-concept research to clinical validation and early-stage implementation. The field is currently entering a new phase in which success will be determined not only by algorithmic performance but also by thoughtful integration into clinical practice.

Looking ahead, future developments will likely include increasingly powerful foundation models, multimodal data integration, and expanded real-world implementation studies. Nevertheless, the fundamental objective remains unchanged: to ensure that artificial intelligence augments human expertise, preserves patient trust, promotes equitable access to care, and ultimately improves visual outcomes for patients worldwide.
